# Therapeutic Targeting of the GSK3β-CUGBP1 Pathway in Myotonic Dystrophy

**DOI:** 10.3390/ijms241310650

**Published:** 2023-06-26

**Authors:** Maggie Lutz, Miranda Levanti, Rebekah Karns, Genevieve Gourdon, Diana Lindquist, Nikolai A. Timchenko, Lubov Timchenko

**Affiliations:** 1 Division of Neurology, Cincinnati Children’s Hospital, Cincinnati, OH 45229, USA; maggie.lutz@cchmc.org (M.L.); miranda@mogen.com (M.L.); 2 Departments of Gastroenterology, Hepatology & Nutrition, Cincinnati Children’s Hospital, Cincinnati, OH 45229, USA; rebekah.karns@cchmc.org; 3 Sorbonne Université, Inserm, institut de Myologie, Centre de Recherche en Myologie, 75013 Paris, France; genevieve.gourdon@inserm.fr; 4 Imagine Research Center, Cincinnati Children’s Hospital, Cincinnati, OH 45229, USA; diana.lindquist@cchcm.org; 5 Department of Pediatrics, University of Cincinnati, Cincinnati, OH 45221, USA; nikolai.timchenko@cchmc.org; 6 Department of Surgery, Cincinnati Children’s Hospital, Cincinnati, OH 45229, USA

**Keywords:** Myotonic Dystrophy (DM1), congenital Myotonic Dystrophy, development of therapy, myotonia, brain atrophy, GSK3β, CUGBP1, *HSA^LR^* mice, DMSXL mice, GSK3 inhibitor

## Abstract

Myotonic Dystrophy type 1 (DM1) is a neuromuscular disease associated with toxic RNA containing expanded CUG repeats. The developing therapeutic approaches to DM1 target mutant RNA or correct early toxic events downstream of the mutant RNA. We have previously described the benefits of the correction of the GSK3β-CUGBP1 pathway in DM1 mice (*HSA^LR^* model) expressing 250 CUG repeats using the GSK3 inhibitor tideglusib (TG). Here, we show that TG treatments corrected the expression of ~17% of genes misregulated in DM1 mice, including genes involved in cell transport, development and differentiation. The expression of chloride channel 1 (*Clcn1*), the key trigger of myotonia in DM1, was also corrected by TG. We found that correction of the GSK3β-CUGBP1 pathway in mice expressing long CUG repeats (DMSXL model) is beneficial not only at the prenatal and postnatal stages, but also during adulthood. Using a mouse model with dysregulated CUGBP1, which mimics alterations in DM1, we showed that the dysregulated CUGBP1 contributes to the toxicity of expanded CUG repeats by changing gene expression and causing CNS abnormalities. These data show the critical role of the GSK3β-CUGBP1 pathway in DM1 muscle and in CNS pathologies, suggesting the benefits of GSK3 inhibitors in patients with different forms of DM1.

## 1. Introduction

Myotonic Dystrophy (DM1) is a multisystemic, genetic disease that affects skeletal muscle, the heart, the CNS and other tissues [1]. The severity of the disease symptoms in patients with DM1 is highly variable, and has an approximate correlation with the length of CTG repeat expansions. Currently, there is no disease-based cure for DM1. While the molecular mechanisms of DM1 have been well described in many studies [2,3], there are obstacles in the development of DM1 therapies, which include the high variability of the DM1 clinical phenotype, under-developed DM1 biomarkers and the variety of potential therapeutic targets due to the complexity of DM1’s molecular pathology. Several potential targets for DM1 therapy have been suggested, including the editing of expanded CTG repeats within the mutant *DMPK* gene [4,5,6,7,8], degradation of the mutant *DMPK* mRNA [9,10,11,12] and the correction of RNA-binding proteins involved in DM1, such as MBNL1 and CUGBP1/CELF1 [13,14,15]. 

Some of the proposed therapeutic approaches to DM1 are still in pre-clinical development, while others are being tested in clinical studies. The use of antisense oligonucleotides (AON) specific to *DMPK* mRNA degraded mutant *DMPK* mRNA in preclinical studies [9,10]. However, the initial application of this approach in the Phase I/II clinical trial for DM1 showed that the drug concentration observed in the skeletal muscles of treated patients was not sufficient to significantly reduce the mutant *DMPK* mRNA, suggesting that the efficiency of AON delivery in skeletal muscle needs to be improved [16]. 

One of the proposed therapeutic targets in DM1, which reached testing in the CDM1 clinical trial, is RNA-binding protein, CUGBP1, which is compromised by mutant CUG repeats [17,18,19]. Using DM1 cell and mouse models, our group showed that mutant CUG repeats not only alter CUGBP1 levels [19], but also change CUGBP1 activity due to an increase in GSK3β kinase; this prevents CUGBP1 phosphorylation at S302 by cyclin D3-CDK4 [20], converting active (phosphorylated at S302) CUGBP1^ACT^ into inactive CUGBP1 repressor CUGBP1^REP^, which is unphosphorylated at S302. The significance of this event in DM1 pathogenesis was demonstrated in preclinical studies showing that the correction of GSK3β using small-molecule GSK3β inhibitors had positive effects on the improvement of skeletal muscle and CNS defects in DM1 mouse models [20,21,22] and in a human study, a Phase II clinical trial for juvenile and adolescent patients with CDM1 [15,23]. Using GSK3 inhibitors, we previously showed that the correction of the GSK3β-CUGBP1 pathway reduces the muscle phenotype in *HSA^LR^* mice [20], a model for adult, classic DM1 expressing CUG repeats in skeletal muscle [24]. Moreover, short-term treatments of young *HSA^LR^* mice (at the early stages of the disease) with the GSK3 inhibitors prevented the development of muscle weakness and reduced muscle histopathology at a later age [20]. The small-molecule GSK3 inhibitor tideglusib (TG) was also beneficial in an DMSXL model, and expresses long CUG repeats (>1000) under the human *DMPK* promoter in skeletal muscle, the heart and the CNS [25]. Short-term TG treatments of young DMSXL mice showed a positive effect on muscle histopathology [22]. However, the effect was stronger when TG was used prenatally or postnatally, significantly improving the survival rate of under-developed homozygous DMSXL mice, increasing their muscle strength and reducing anxiety, measured using the open field test, at a young age [22]. 

The first goal of this study was to determine the effect of TG treatments on global gene expression in the skeletal muscles of *HSA^LR^* mice to investigate pathways that are corrected by 2-week treatment with TG. The second goal was to examine whether TG treatments can correct muscle weakness in adult homozygous DMSXL mice that survived under-development and postnatal stress. In our early preclinical study, we focused mainly on prenatal and postnatal treatments of DMSXL mice because about 60% of homozygous DMSXL mice die during the first month of life [25]. Postnatal homozygous DMSXL mice are very weak due to, at least in part, delayed development. We thought that surviving homozygous DMSXL mice that overcame under-developmental stress might be less responsive to the correction of muscle weakness, mediated by TG, during adulthood. To address this question, this study analyzed the effect of TG on muscle weakness in surviving adult homozygous DMSXL mice. Since inactive un-ph-S302-CUGBP1 (CUGBP1^REP^) is a part of the TG-GSK3β pathway, we also continued the analysis of the contribution of CUGBP1^REP^ to CNS dysfunction in DMSXL mice using the open field test and brain MRI analysis. Our new data, together with the previously published studies, expand our knowledge about the benefits of the TG-GSK3β-CUGBP1 pathway in DM1 mouse models, suggesting that GSK3 inhibitors could be effective in DM1 patients of different ages, including pediatric, juvenile and adult patients with DM1.

## 2. Results

### 2.1. RNA-Seq Approach Revealed That TG Partially Corrects Biological Processes in HSA^LR^ Muscle Associated with Cell Transport, Development and Differentiation

We have previously reported that the treatment of adult *HSA^LR^* mice with TG for two weeks (0.1 μg/g, two times a week) resulted in significant improvement in skeletal muscle histopathology, the correction of muscle atrophy and the normalization of grip strength [22]. An early study of the benefits of GSK3 inhibitors showed that the treatment of adult *HSA^LR^* mice with the GSK3 inhibitor lithium reduced myotonia [20]. Moreover, myotonia remained reduced in the treated mice several weeks after the completion of the treatments. Such a strong effect of the inhibitors of GSK3β on the improvement of skeletal muscle pathology in *HSA^LR^* mice suggests that the inhibitors of GSK3 might correct critical biological processes important for muscle function that are misregulated by toxic CUG repeats. To investigate epigenetic pathways that might be improved by TG treatments, we performed global gene expression analysis in the age- and gender-matched skeletal muscles (gastrocnemius (gastroc)) from adult (6 months of age, males) WT and *HSA^LR^* mice treated with the vehicle or TG for two weeks (0.1 μg/g, two times a week) using RNA-seq analysis. Genes with statistically significant changes of at least 1.5-fold or greater were selected for the study. We found that 667 genes, including up-regulated and down-regulated genes, were changed ≥1.5 fold in the control labrasol-treated *HSA^LR^* muscle vs. WT muscle (Table 1). TG treatments (two weeks, two times a week) partially normalized ~17% of genes altered in the *HSA^LR^* muscle (including up- and down-regulated genes) (Table 1). We think that if the duration of the treatments is increased, this might result in a larger number of corrected genes.

The most significantly altered biological processes in the skeletal muscles of the vehicle-treated *HSA^LR^* mice include system development (*p* = 6.3 × 10^−7^), the regulation of programmed cell death (*p* = 2.2 × 10^−6^), cell differentiation (*p* = 2.7 × 10^−6^), regulation of the apoptotic process (*p* = 2.8 × 10^−6^) and anatomic structure development (*p* = 3.4 × 10^−6^). 

Our search for the enriched down-regulated processes in *HSA^LR^* muscle identified a list of processes, which includes those associated with the regulation of hormone levels (*p* = 1.47 × 10^−7^), the regulation of transport (*p* = 1 × 10^−6^), secretion (1.26 × 10^−6^), synaptic vesicle localization (*p* = 1.5 × 10^−6^), the synaptic vesicle cycle (*p* = 1.75 × 10^−6^), system development (*p* = 1.8 × 10^−6^), secretion by cells (3.88 × 10^−6^), the developmental process (*p* = 6.39 × 10^−6^) and cell differentiation (*p* = 3.89 × 10^−5^) (Table 2). 

The enriched processes up-regulated in control *HSA^LR^* muscle include pathways linked to cell death (the regulation of programmed cell death (*p* = 2.41 × 10^−10^); the regulation of apoptotic processes (*p* = 3.96 × 10^−10^), the negative regulation of apoptotic processes (*p* = 1.32 × 10^−8^) and the negative regulation of cell death (*p* = 2.19 × 10^−8^)) and the regulation of cell proliferation (*p* = 1.24 × 10^−7^).

Given the correction of muscle atrophy, the grip strength and myotonia in *HSA^LR^* mice treated with GSK3 inhibitors, we first asked whether genes that are involved in the regulation of transport, cell development and cell differentiation in skeletal muscle are corrected by TG. As shown (Table 2), the expression of 10 genes out of 93 misregulated genes linked to the regulation of transport were improved by TG. Remarkably, the list of TG-corrected genes involved in the regulation of transport includes chloride ion channel 1 (*Clcn1*), associated with the development of the main muscle symptom of DM1, myotonia. 

**Figure 1:**
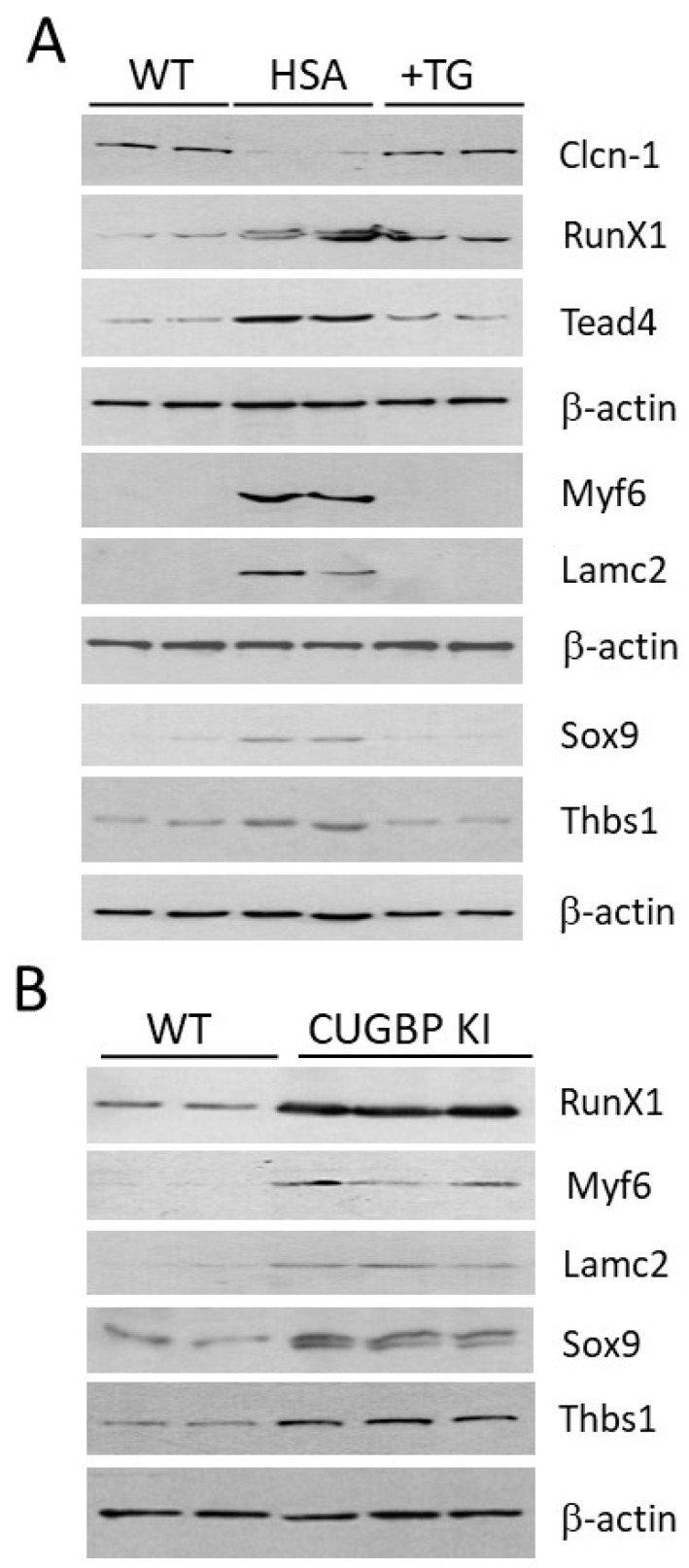
(**A**). Western blot analysis of the selected genes that are altered in skeletal muscles of *HSA^LR^* mice, and which are improved by the TG treatments. Two matched (6-month-old males) WT mice, two vehicle-treated *HSA^LR^* mice and two *HSA^LR^* mice treated with TG for 2 weeks were analyzed. Separate β-actin images as controls are presented for *Clcn1*, RunX1 and Tead4; Myf6 and Lamc2; and Thbs1 and Sox9. (**B**). Selected genes altered in skeletal muscles of *HSA^LR^* mice are similarly changed in skeletal muscles of CUGBP1 KI mice. Two matched (3-month-old males) WT and three homozygous CUGBP1 KI mice were examined. B-actin is the control for protein loading.

Normally, *Clcn1* transports chloride ions, which regulate nerve and muscle cell membrane excitability. As is already known, *Clcn1* is mis-spliced and down-regulated in the *HSA^LR^* muscle and in the skeletal muscles of patients with DM1 [27,28]. The mis-splicing of and reduction in *Clcn1* are associated with the development of myotonia in DM1. We found that the expression of *Clcn1* was nearly corrected in the TG-treated *HSA^LR^* mice. The immunoanalysis confirmed the correction of *Clcn1* in the skeletal muscles of the TG-treated *HSA^LR^* mice (Figure 1A). 

Thus, an un-biased RNAseq approach, and subsequent Western blot analysis, confirmed our previous finding that the inhibition of GSK3 is linked to the reduction in myotonia [20] caused by CUG repeats in *HSA^LR^* mice, via the correction of *Clcn1* levels. The list of genes associated with transport corrected by TG in *HSA^LR^* muscle includes a transcription factor, *Runx1*, that regulates several ion channels, such as sodium channel type V (Scn5a), amiloride-sensitive cation channel 1 (Accn1) and cholinergic receptor gamma subunit (Chrng) [29]. In addition, Runx1 regulates signaling molecules, such as phospholamban (Pln), and important muscle proteins, including embryonic myosin heavy chain (myh3) and myosin heavy chain IIA (Myh2). It has been shown that the misregulation of these genes in the skeletal muscles of *Runx1*-deficient mice causes dramatic muscle wasting [29]. Runx1 is also important for the maintenance of muscle integrity as it prevents denervated myofibers from undergoing myofibrillar disorganization and autophagy, found in congenital myopathies. Since Runx1 is increased in muscles after denervation, it might play a compensatory role, preventing muscle wasting. 

Besides *Runx1*, TG normalized several critical transcription factors misregulated in *HSA^LR^* muscle, including *Tead4* and *Myf6*, which are also up-regulated in denervated muscle atrophy [30]. TEAD4 belongs to a family of transcription factors that bind a muscle-specific cytidine–adenosine–thymidine element (MCAT) in the gene regulatory regions, promoting myogenesis [31]. Myogenic factor 6 (Myf6), also known as MRF4 or herculin, is a basic helix–loop–helix (bHLH) transcription factor, which is involved in the later stages of myogenesis [32]. The correction of these genes in TG-treated *HSA^LR^* muscle likely contributes to the reduction in myopathy and decrease in muscle atrophy. Sox9, a transcription factor important for musculoskeletal development that is expressed in muscle progenitor cells [33], was also improved by TG treatments (Table 2, Figure 1A).

Other critical genes corrected by TG treatment encode several regulatory proteins that are involved in muscle growth and muscle regeneration. A receptor of Wnt7a, Frizzle 7 (*Fzd7*), is reduced in *HSA^LR^* muscle. TG treatments improved *Fzd7* expression. Wnt7a is a component of the Wnt-β-catenin pathway, and is important for cell growth and proliferation. One of the functions of Wnt7a-Fzd7 is the symmetric expansion of myogenic satellite cells during muscle cell repair [34]. In differentiated myofibers, Wnt7a binding to Fzd7 directly activates the Akt/mTOR pathway, inducing fiber hypertrophy. Thus, correction of *Frz7* in *HSA^LR^* muscle might contribute to the improvement of muscle regeneration and fiber growth in TG-treated *HSA^LR^* mice. TG also corrected the expression of several genes associated with system development, developmental processes and cell differentiation pathways (*Lamc2, Tnfrsf12* and *Thbs1)*. Laminin subunit gamma-2 (Lamc2) is an extracellular matrix protein that belongs to laminins, a major component of the basal lamina. Laminins play a critical role in cell differentiation, migration and adhesion [35]. Tnfrsf12 (TNF receptor superfamily member 12) is a receptor for TWEAK cytokine, which induces muscle atrophy [36]. 

We found that *Thbs1* (Thrombospondin 1) is increased in *HSA^LR^* muscle, and TG normalizes *Thbs1* levels. THBS1 is a member of the THBS glycoproteins, which mediate cell–cell and cell–matrix interactions [37]. It binds to several proteins, including integrin, fibrinogen, collagens and TGF-β [37]. Thbs1 is increased in response to a high-fat diet (HFD) and may induce insulin resistance [38]. Respectively, the deletion of *Thbs1* in mice protects them from insulin resistance and from muscle fibrotic damage in response to HFD. Thus, the increase in *Thbs1* in *HSA^LR^* muscle suggests that *HSA^LR^* mice have metabolic abnormalities and that the treatment with TG corrected these alterations. In addition to the improvement of *Clcn1*, Western blot analyses confirmed the improvement of protein levels of Runx1, Tead4, Myf6, Lamc2, Sox9 and Thbs1 in the skeletal muscles of *HSA^LR^* mice treated with TG (Figure 1A).

It was shown that the IGF1 levels are in the lower normal range in DM1 patients and that the delivery of human IGF1 or human IGF1/human IGFBP-3 increases lean body mass and improves metabolism in patients with DM1 [39,40]. IGF1 is also reduced in DMSXL muscle [25]. We found that the *IGF1* and IGF1 substrate, *IRS*-1, is reduced in *HSA^LR^* muscle. However, there was no positive effect of TG on these mRNAs in the treated mice, despite the previous report that TG has a positive effect on IGF-1 [41]. This could be due to insufficient TG doses or the short duration of the treatment (two weeks).

One of the important questions related to the benefits of TG treatments in congenital DM1 (CDM1) and DM1 is whether TG corrects muscle pathology mainly via the GSK3β-cyclin D3-CUGBP1 pathway or whether TG normalizes other GSK3β-dependent substrates in *HSA^LR^* muscle. To answer this question, we examined whether the genes altered in *HSA^LR^* muscle and corrected by TG are similarly misregulated in the skeletal muscles of the mutant mice expressing inactive CUGBP1^REP^ with the mutated site for GSK3β-cyclin D3-CDK4 phosphorylation, S302A-CUGBP1 (S333A-CUGBP1 in mice) [26]. As shown (Figure 1B), Runx1, Myf6, Lamc2, Sox9 and Thbs1 are also up-regulated in the skeletal muscles of S302A-CUGBP1 KI mice, as in *HSA^LR^* mice (Figure 1A). Thus, several significantly altered biological processes associated with cell transport, development and differentiation are partially corrected in the TG-treated *HSA^LR^* muscle. At least some altered genes in *HSA^LR^* muscle are corrected via the normalization of CUGBP1 activity. 

### 2.2. Grip weakness Is the Main Feature of Surviving Homozygous DMSXL Mice up to 1 Year of Age

It has been found that juvenile and adolescent CDM1 patients treated with TG in the Phase II clinical trial showed improvement in CNS defects and muscle pathology, including myotonia and fatigue; however, more studies are needed to address the effect of TG treatments on muscle weakness in adult CDM1 patients. Therefore, we examined the effect of TG on grip strength in adult homozygous DMSXL mice that survived postnatal stress. 

**Figure 2:**
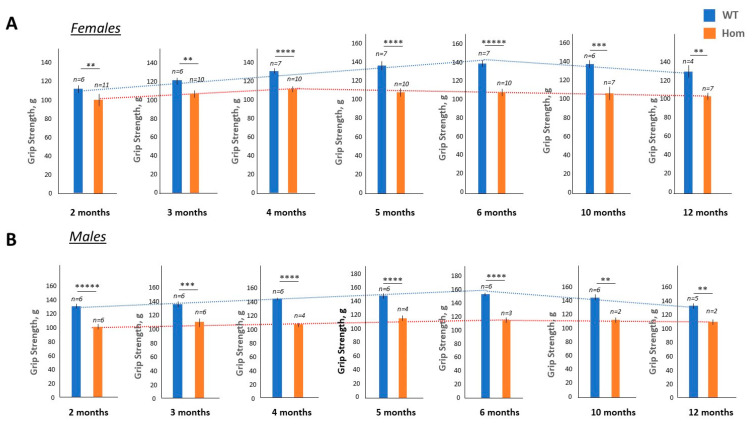
Grip strength analysis of the matched WT and homozygous DMSXL mice ((**A**) females and (**B**) males) from 2 to 12 months of age. The number of mice in each group is shown at the top. **, ***, **** and ***** are *p* values of <0.01, 0.001, 0.0001 and 0.00001, respectively.

We have previously shown that postnatal homozygous DMSXL mice remain weak during the first months of life and that grip weakness is a good parameter to monitor the effect of TG treatments in young DMSXL mice after prenatal or postnatal therapy [22]. Therefore, prior testing the effectiveness of TG treatments in adult homozygous DMSXL mice, we investigated whether surviving homozygous DMSXL mice remain weak during their life span. Examination of the grip strength in the matched WT and homozygous DMSXL mice from 2 to 12 months of age revealed that WT mice (females) show an increase in grip strength from 2 to 6 months, while older WT mice (10 and 12 months) have slightly reduced grip strength, likely due to aging (Figure 2A). 

In contrast, matched homozygous DMSXL mice (females) had grip strength that remained almost at the same level over time, and their grip strength was significantly reduced relative to the WT mice. Similarly, a steady reduction in grip strength relative to the matched WT mice was observed in the male group of homozygous DMSXL mice from 2 to 12 months of age (Figure 2B). There was no progression of grip weakness in homozygous DMSXL mice with age. These data show that the surviving homozygous DMSXL mice remained weak over 12-month period. Therefore, grip weakness could be used as an outcome to monitor the treatment of young and adult homozygous DMSXL mice of both genders with TG or with other therapeutics.

### 2.3. Chronic Treatments with TG Restore Muscle Strength in Adult Homozygous DMSXL Mice

**Figure 3:**
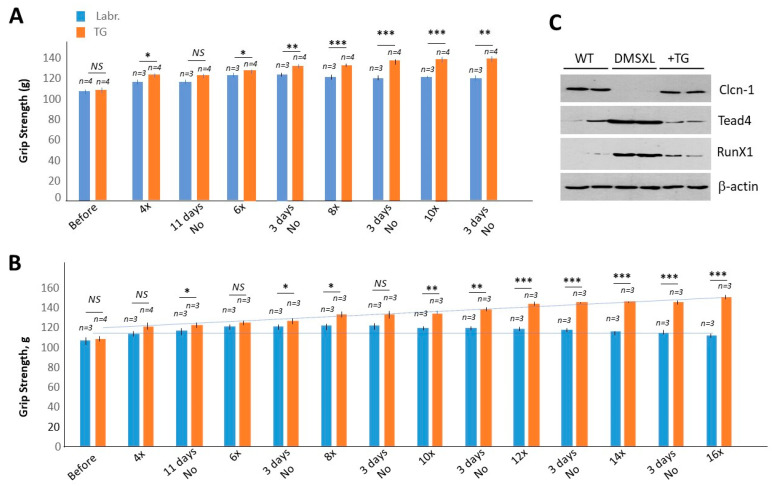
Comparison of the grip strength of homozygous DMSXL mice treated with TG vs. vehicle ((**A**) females and (**B**) males). The number of mice per group is shown at the top. The number of TG doses from the beginning to the end of the treatment (10 doses (10×) in the female group and 16 doses (16×) in the male group) is shown at the bottom. After 4 doses of TG, both mouse groups were maintained without treatments for 11 days. After 6, 8, 10, 12, 14 and 16 doses of TG, treated mice were kept for 3 days without treatments. *, ** and *** are *p* values of <0.05, 0.01 and 0.001, respectively. (**C**) Correction of *Clcn1*, Runx1 and Tead4 in muscle of DMSXL mice treated with TG.

Since homozygous DMSXL survivors are characterized by grip weakness during adulthood, we examined the effect of TG on grip weakness in 3-month-old homozygous DMSXL mice. Four groups of 3-month-old homozygous DMSXL mice of both genders were treated with TG (0.1 μg/g) or a vehicle (labrasol). As we described previously, in adult *HSA^LR^* mice, normal levels of GSK3β were observed after treatment with two doses of TG analog, TDZD8 [20]. To achieve a positive effect in prenatal and postnatal homozygous DMSXL mice, two–three doses of TG were sufficient [22]. Therefore, we initially treated 3-month-old homozygous DMXSL mice two times a week for two weeks. We found that after two weeks of treatment, the grip strength was significantly increased in the TG-treated mice (females) relative to the vehicle-treated group (Figure 3A). 

The improved grip strength remained at the same level for the following 11 days without treatments. Continuous treatment of this group of homozygous DMSXL mice with TG for an additional 3 weeks (two times a week, 0.1 μg/g) showed further significant improvement in grip strength by ~27% relative to the values obtained prior to the initiation of treatment. The grip strength was also increased in the naturally growing vehicle-treated homozygous DMSXL mice (females), but by only ~16% relative to the baseline (prior to the initiation of the treatment). Thus, the TG-treated 3-month-old homozygous DMSXL mice (females) show about an 11% improvement in grip strength over the matched vehicle-treated mice after 5 weeks of treatment.

An even better result was observed in the male group of 3-month-old homozygous DMSXL mice treated with TG (Figure 3B). The initial treatment of this group with the same dose of TG (0.1 μg/g) for 2 weeks (2 times a week) showed about an 11% increase in grip strength in the TG-treated group, while the grip strength was increased by only ~6% in the vehicle-treated group. The grip strength remained about the same in the treated mice for the next 11 days without treatment. However, the following treatment for 6 weeks showed that the grip strength in the TG-treated homozygous DMSXL mice returned to normal values, increasing by almost 39% compared to the grip strength prior to treatment. The increase in grip strength in the vehicle-treated matched homozygous DMSXL mice was only ~5%. Thus, the grip strength was significantly improved (by about 34%) in the TG-treated adult homozygous DMSXL mice vs. the matched vehicle-treated mice. These results show that the TG treatments of adult homozygous DMSXL mice were effective and completely restored muscle strength. While the average grip strength in 5-month-old WT mice (males) was 149.96 g (*n* = 6) (Figure 2B), the average grip strength in the TG-treated 5-month-old DMSXL mice (males) after the completion of the TG treatments was 150.3 g (*n* = 3) (Figure 3B). Similar results were obtained in homozygous DMSXL mice of both genders. We noticed that the body weights of homozygous DMSXL mice in the control and TG-treated groups were almost unchanged. This contrasts with the strong positive effect of TG on total body weight after prenatal treatments of homozygous DMSXL mice [22]. This could be because the effect of TG on body weight is stronger during development than in adulthood. 

Since homozygous DMSXL mice are prone to dying during their life span, we carefully monitored the possible negative effects of the TG treatments on the survival rate in the treated DMSXL mice. Although one of the TG-treated homozygous DMSXL mice (males) was losing weight and was sacrificed prior the completion of the course of the treatments, the weight loss was found to be unrelated to the treatment because the mouse had abnormally grown teeth which likely interfered with food intake. 

We also examined the expression of several genes that are altered in the *HSA^LR^* muscle of DMSXL mice. We found that *Clcn1* is reduced, while Runx1 and Tead4 were increased, in the gastroc of the labrasol-treated DMSXL mice (Figure 3C). It is important that TG treatments of DMSXL mice correct the expression of *Clcn1*, Runx1 and Tead4. These findings suggest that genes that are important for muscle function, such as *Clcn1*, Runx1 and Tead4, are similarly altered by CUG repeats in *HSA^LR^* and DMSXL muscles and that TG corrects the expression of these genes. Thus, we found that the TG treatments were beneficial for adult homozygous DMSXL survivors and that the 5–8-week treatments led to the complete recovery of muscle strength. We are currently examining the effects of TG treatments on the CNS phenotype in DMSXL mice. While grip strength was completely recovered in the TG-treated homozygous DMSXL mice, the open field test values are very variable, suggesting the need to increase the number of analyzed mice.

### 2.4. The Contribution of the Abnormal Activity of CUGBP1 to Cognitive Dysfunction in DM1

**Figure 4:**
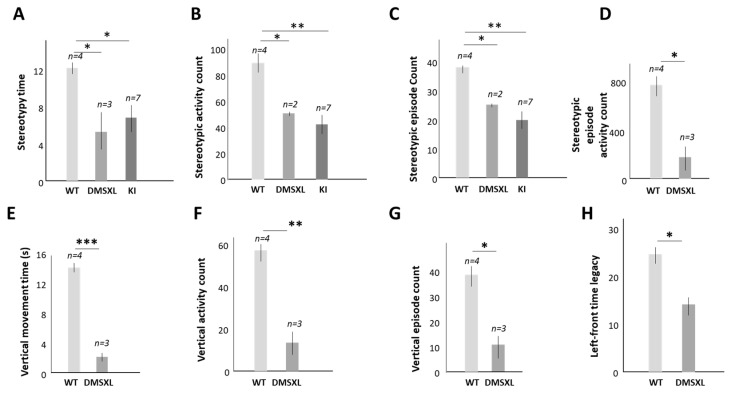
The stereotypy time (**A**), stereotypic activity counts (**B**) and stereotypic episode count (**C**) are reduced in the matched homozygous DMSXL and CUGBP1 KI mice vs. WT mice (males, 1 month). Open field test was performed with naive mice for 5 min. Homozygous DMSXL mice (males, 1 month) also show reduced stereotypic episode activity count (**D**), vertical movement time (**E**), vertical activity count (**F**), vertical episode count (**G**) and left-front time legacy (**H**) relative to matched WT mice. The number of mice is shown at the top. *, ** and *** are *p* values of <0.05, 0.01 and 0.001, respectively.

While the majority of our studies were focused on the effect of GSK3 inhibitors on muscle pathology in DM1, each form of DM1 is also associated with cognitive defects [42,43,44]. CDM1 is characterized by mild-to-severe intellectual disability, delayed motor development, autism and attention deficit/hyperactivity disorder (ADHD). In childhood DM1, CNS symptoms include learning difficulties and mood disorders. The juvenile form of DM1 is characterized by abnormal social communication and behavioral problems. Patients with adult DM1 have problems with executive function and visuospatial ability. It has been reported that TG treatments showed promising results in patients with CDM1 that reduces cognitive dysfunction [23]. Prenatal treatments of DMSXL mice with TG also had a positive effect on the CNS disorder, reducing anxiety, as shown by the increase in the center distance travelled by the treated mice in the open field test [22]. The TG treatments of DMSXL mice restored active CUGBP1 in the brain, suggesting that abnormal activity of CUGBP1 contributes to CNS disorder in DM1 and CDM1. To determine the contribution of CUGBP1^REP^ to cognitive dysfunction in CDM1 and DM1, we began behavioral analysis of S302A-CUGBP1 KI mice, in which CUGBP1 was un-phosphorylatable at S302 by the GSK3β-CDK4 pathway [26]. We found that both CUGBP1 KI and DMSXL mice were characterized by reduced stereotypic activities, as shown by the reduced stereotypy time, reduced stereotypic activity counts and reduced stereotypic episode counts (Figure 4A–C). 

Some other behavioral features differed in CUGBP1 KI and DMSXL mice. For instance, only young DMSXL mice showed reduced stereotypic episode activity counts and a decrease in several other parameters, such as vertical movement time, vertical activity counts, vertical episode count and left-front time legacy (Figure 4D–H). 

### 2.5. Magnetic Resonance (MRI) and Diffusion Tensor Imaging (DTI) Studies of CUGBP1 KI and DMSXL Mouse Brains

**Figure 5:**
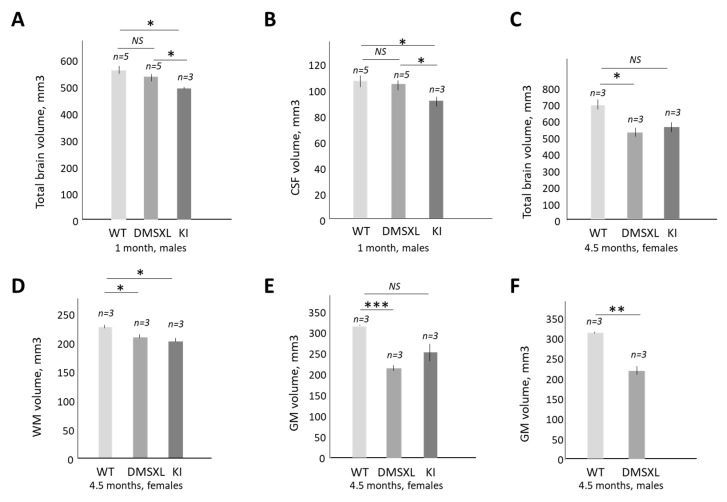
The reduction in total brain (**A**) and CSF (**B**) volumes in 1-month-old homozygous CUGBP1 KI mice vs. matched WT or homozygous DMSXL mice (males) is shown. (**C**) Comparison of the total brain volume in the matched 4.5-month-old WT, homozygous CUGBP1 KI and DMSXL mice (females). (**D**) WM volume is similarly reduced in adult homozygous CUGBP1 KI and DMSXL mice relative to WT mice. (**E**) Comparison of the GM volume in the matched adult WT, DMSXL and CUGBP1 KI mice (females). (**F**) The GM volume is strongly reduced in adult homozygous DMSXL mice (males) vs. matched WT mice. The number of mice per group is shown at the top. Standard deviations are also shown. *, ** and *** are *p* values of <0.05, 001 and 0.001, respectively.

It is known that cognitive defects in DM1 are accompanied by morphological changes in the brain [45]. To determine whether the increase in inactive CUGBP1^REP^ affects brain volume in CUGBP1 KI and in DMSXL mice, we performed brain volumetric analysis of these mice via MRI. Two age groups (1 month and 4.5 months) of matched WT, CUGBP1 KI and DMSXL mice were analyzed. We found a significant reduction (~10%) in total brain volume in 1-month-old homozygous CUGBP1 KI mice relative to matched WT mice, while the total brain volume was not changed in 1-month-old DMSXL mice (Figure 5A).

In addition, the volume of Cerebral Spinal Fluid (CSF) was reduced in 1-month-old CUGBP1 KI mice (Figure 5B). No significant changes in the CSF volume were identified in the matched DMSXL mice. These data show that the accumulation of inactive CUGBP1^REP^ in homozygous CUGBP1 KI mice caused a reduction in brain volume at a young age. A similar analysis showed that adult (4.5 months) homozygous DMSXL mice were characterized by a strong loss of total brain volume (~23%) vs. matched WT mice (Figure 5C). The matched adult CUGBP1 KI mice also showed a ~20% reduction in total brain volume; however, this value is not significant, likely due to the variability of the values between mice. Thus, it appears that there is possible loss of brain volume in homozygous DMSXL mice in adulthood. Examination of the white matter (WM) volume showed that while no significant volumetric changes were observed in young CUGBP1 KI and DMSXL mice, there was a significant reduction in the WM volume in both adult DMSXL and CUGBP1 KI mice (Figure 5D), by approximately 8% in DMSXL brains and 11% in CUGBP1 KI brains. The grey matter (GM) volume was even more strongly reduced in adult DMSXL mice (by ~30%) vs. WT mice (Figure 5E). The GM volume was also reduced by ~19% in adult CUGBP1 KI mice; however, this change was not significant. The GM volume reduction in DMSXL mice was independent of gender because adult homozygous DMSXL mice (males) also showed about a 30% reduction in GM volume (Figure 5F), like the matched DMSXL females (Figure 5E). Taken together, these findings show that the increase in inactive CUGBP1^REP^ due to the mutation of the GSK3β-CDK4 phosphorylation site in CUGBP1, and/or due to the expression of the mutant CUG repeats in DMSXL mice [22], causes an approximately comparable reduction in WM volume. 

**Figure 6:**
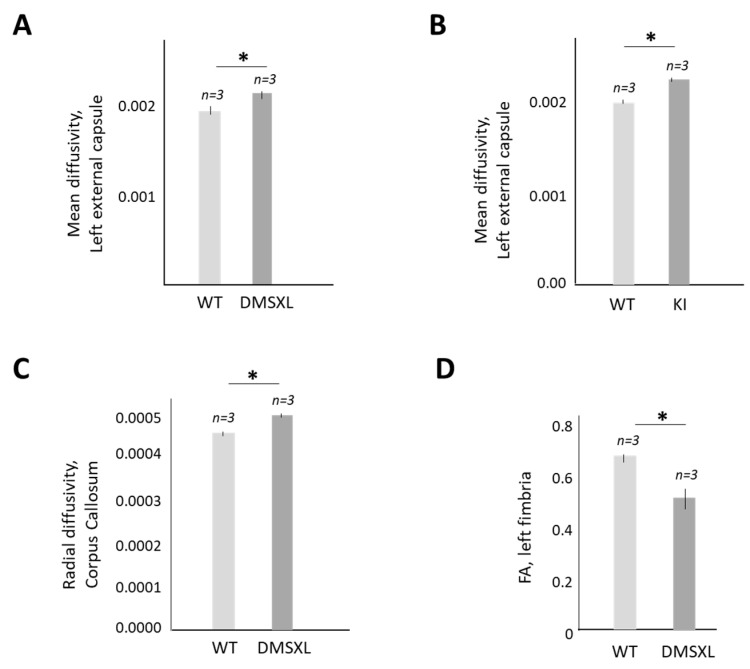
Mean diffusivity is increased in left external capsule in homozygous DMSXL (**A**) and CUGBP1 KI mice (**B**) vs. matched WT mice. In (**A**), two groups of matched 4.5-month-old WT and DMSXL mice (males) were used, while in (**B**), two groups of WT and DMSXL mice (females) were examined. (**C**) Radial diffusivity is increased in corpus callosum of 4.5-month-old homozygous DMSXL mice (males) vs. matched WT mice. (**D**) Fractional anisotropy is reduced in left fimbria of 4.5-month-old DMSXL mice (males). Three mice per group were analyzed. Standard deviations are shown. * *p* value < 0.05.

The status of the WM in the CUGBP1 KI and DMSXL brains was examined using DTI imaging. As shown (Figure 6A,B), the mean diffusivity is significantly increased in left external capsules in 4.5-month-old DMSXL and CUGBP1 KI mice vs. matched WT mice. 

The increase in the mean diffusivity suggests a reduction in WM integrity in the brains of DMSXL and CUGBP1 KI mice due to myelin or axonal degradation. In support of this, radial diffusivity was also increased in the corpus callosum in 4.5-month-old DMSXL mice (males) vs. WT mice (Figure 6C), while fractional anisotropy (FA) was reduced in the left fimbria (Figure 6D). Thus, the increase in CUGBP1^REP^ in CUGBP1 KI mice, as well as in the expression of the mutant CUG repeats (which also increases CUGBP1^REP^ in DMSXL mice) [22], causes a significant loss of brain volume and appears to compromise the integrity of the WM. 

### 2.6. Increase in CUGBP1^REP^ in DMSXL Mice Further Worsens the Phenotype of DMSXL Mice

To determine the contribution of CUGBP1^REP^ to the phenotype of DMSXL mice, we generated a double-mutant mouse line in which heterozygous DMSXL mice were crossed with heterozygous CUGBP1 KI mice. The analysis of the frequency of mice with different genotypes in the double-mutant line (23 families) showed that the double-mutant heterozygous (hetCUGBP1-KI-hetDMSXL) mice represented ~24.1% (Table 3). 

**Figure 7:**
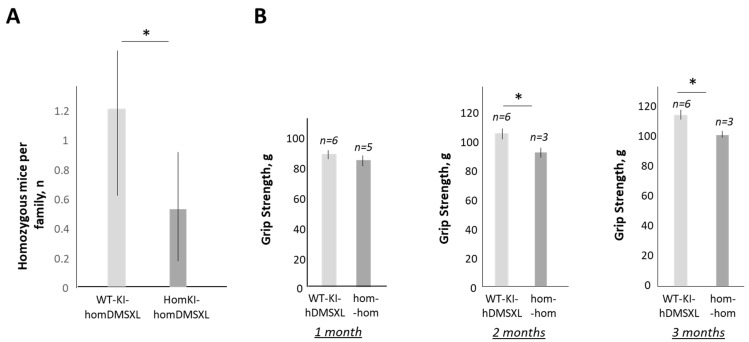
The effect of CUGBP1^REP^ on the phenotype of DMSXL mice in the double-mutant CUGBP1-KI-DMSXL mouse strain. (**A**) The number of double-mutant homozygous mice per family is reduced vs. single-mutant DMSXL mice in CUGBP1-KI-DMSXL strain. A total of 23 families containing 232 mice were analyzed. * *p* value < 0.05. (**B**) Grip strength comparison of the single-mutant WT-CUGBP1-KI-homDMSXL mice and double-mutant homozygous mice. The matched mice (females) at 1–3 months of age were compared. The number of analyzed mice is shown at the top. * *p* value < 0.05.

A comparison of the percentages of single-mutant WT-CUGBP1-KI-homDMSXL mice and double-mutant homCUGBP1-KI-homDMSXL mice showed that the percentage of the double-mutant homozygous mice was ~2.3 times lower (5.2%) vs. the single-mutant WT CUGBP1-KI-homDMSXL (12.1%). It is known that homozygous DMSXL mice die during the first month of life [22,25]. We found that the survival rate of the single-mutant homDMSXL mice in this strain was ~71.4% (Table 3). However, the survival rate of the double-mutant homozygous mice was only ~41.7% (Table 3). Thus, the increase in CUGBP1^REP^ in DMSXL mice has a negative effect on the development of DMSXL mice. Respectively, the number of double-mutant homozygous mice per family was significantly reduced relative to single-mutant homozygous DMSXL mice (Figure 7A). 

The analysis of the grip strength of the double-mutant mice showed that the grip strength was significantly reduced in the double-mutant homozygous mice vs. single-mutant WT-CUGBP1-KI-homDMSXL mice (Figure 7B). These data confirm that the increase in CUGBP1^REP^ in homozygous DMSXL mice further worsens the phenotype of DMSXL mice.

## 3. Discussion

This study provides several important findings related to the development of DM1 therapy, based on the correction of GSK3β-CUGBP1 signaling. First, we found that ~17% of genes altered by mutant CUG repeats in the skeletal muscles of *HSA^LR^* mice, including genes connected to cell transport, development and differentiation, are improved by 2-week treatments with TG. One of the corrected genes in the muscle of *HSA^LR^* mice, which is involved in the control of the electric excitability of the skeletal muscle membrane, is *Clcn1*. This critical finding is supported by our previous data showing that the GSK3 inhibitor lithium causes a reduction in myotonia in *HSA^LR^* mice [20]. Myotonia in DM1 is attributed to alteration of the splicing of *Clcn1* and a reduction in *Clcn1* [26,27]. In this paper, we describe that the correction of GSK3β in *HSA^LR^* mice normalizes *Clcn1* levels in skeletal muscle (Figure 1A). This positive effect is likely due to a reduction in mutant CUG repeats, since previously, we showed that TG reduces mutant CUG RNA in *HSA^LR^* muscle [22]. Thus, myotonia as well as muscle weakness in *HSA^LR^* mice are corrected by GSK3 inhibitors. It is important that *Clcn1* protein levels were also corrected in the muscle of DMSXL mice treated with TG (Figure 3C).

It is not surprising that TG corrected genes related to fiber growth and cell differentiation. Among them is the receptor of Wnt7a, Frizzle 7 (*Fzd7*), Myf6 and TEAD4. Improvement in the expression of these genes might contribute to the correction of muscle atrophy in TG-treated *HSA^LR^* mice [22]. RNA-Seq analysis also identified the circulating protein, Tbst1, corrected by TG treatments in *HSA^LR^* mice. We are planning to investigate this protein as a potential blood biomarker to monitor the efficacy of GSK3 inhibitors applied in DM1 treatments in clinical trials. 

We also found that the TG treatments are effective for improving muscle strength not only in prenatal or postnatal homozygous DMSXL mice, but also in adult homozygous DMSXL mice (Figure 3). Our previous findings demonstrated that if the inhibitors of GSK3 are applied at the early stages of the disease, such as in prenatal DMSXL mice or in young *HSA^LR^* mice, prior to the development of strong muscle pathology, the inhibitors have long-term positive effects [21,22]. It was unknown whether TG is efficient in adult homozygous DMSXL mice that have survived pre- or postnatal under-development. As shown in this manuscript, the increase in TG treatment duration in adult homozygous DMSXL mice fully restores grip strength to normal levels (Figure 3). These findings suggest that muscle weakness could be corrected by the inhibitors of GSK3 in adult patients with CDM1 and in juvenile DM1. 

It remains to be studied whether other symptoms such as CNS abnormalities and cardiac defects are corrected in adult DMSXL mice treated with TG. While grip strength measures could be successfully used to monitor the effects of TG treatments in DMSXL mice, more work is needed to determine parameters that could be used to monitor the correction of CNS abnormalities. One of the simplest options is the use of the open field test (OFT); however, we observed very variable results in both the vehicle- and TG-treated DMSXL mice, making it difficult to correlate them with the treatments using a relatively small number of homozygous DMSXL mice per group. 

One of the critical results of this study is that homozygous DMSXL mice develop brain atrophy with WM and GM lesions. Thus, the effects of the GSK3 inhibitors and other therapeutics on CNS defects in DMSXL mice could be monitored using DTI imaging. However, more data should be collected to determine whether homozygous DMSXL mice, especially mice with severe under-development at a young age, tolerate the DTI procedure. In our study, homozygous DMSXL mice were sacrificed after DTI testing for histological and molecular analyses. 

It has been shown that CUGBP1^REP^ (de-ph-S302-CUGBP1) is elevated in myoblasts from patients with DM1 [18,19]. This study describes CUGBP1^REP^ as a critical component of the GSK3β-CUGBP1 signaling pathway in DM1 pathogenesis. We have previously found that CUGBP1^REP^ is increased by mutant CUG repeats in the skeletal muscles of *HSA^LR^* mice [20] and in the brains of DMSXL mice [22]. Respectively, the treatments of these mice with the inhibitors of GSK3 converted CUGBP1^REP^ in CUGBP1^ACT^, restoring normal CUGBP1 activity [20,22]. The analysis of the brain morphology and behavioral features of CUGBP1 KI and DMSXL mice showed some similarities in both models (Figure 4, Figure 5 and Figure 6). It is important that both mouse models were characterized by brain atrophy (Figure 5), showing that the GSK3β-CUGBP1 pathway plays a critical role in the development of brain atrophy in DM1. Respectively, the correction of this pathway with the inhibitors of GSK3 should reduce CNS abnormalities. 

In addition, CUGBP1^REP^ contributes to the reduced survival rate of homozygous DMSXL mice, since the number of surviving double-mutant homozygous (homCUGBP1-KI-hom-DMSXL) mice was less than that of single-mutant WT-CUGBP1-KI-homDMSXL mice. The increase in CUGBP1^REP^ also led to a reduction in the percentage of double-mutant homCUGBP1-KI-homDMSXL mice per family relative to single-mutant WT-CUGBP1-KI-homDMSXL mice. This result is not surprising because CUGBP1 has an important function in development. As known, the deletion of *Cugbp1* in mice affects development, leading to reduced body weight in homozygous *Cugbp1* knockout mice and their death at birth or shortly after birth [21]. Thus, the correction of GSK3β-CUGBP1 signaling in DM1 mouse models recovers not only muscle dysfunction (muscle weakness, myotonia, atrophy and myopathy), but might also reduce CNS pathology. Although more pre-clinical work is needed to determine whether the inhibitors of GSK3 reduce brain atrophy in homozygous DMSXL mice, this study shows that the correction of CUGBP1 activity via GSK3β signaling is a critical target in DM1 therapy. 

Additional questions that remain to be answered in pre-clinical studies using TG include those on the effectiveness of TG treatments in old homozygous DMSXL mice and those on whether the increase in the duration of the treatments in older animals is safe. There are some expected difficulties in human clinical studies (regardless of the therapeutics) which might be connected to the evaluation of the effectiveness of the treatments (selection of the quantitative measures and quantification of proper biomarkers) in DM1 populations with varying age and different phenotype complexity. Despite these potential difficulties, DM1 clinical studies are in progress. It would be interesting to see whether the correction of the TG-GSK3β-CUGBP1 pathway in a larger group of CDM1 patients is beneficial. 

## 4. Materials and Methods

### 4.1. Mice and Treatments

DMSXL mice expressing >1000 CTG repeats in the human *DMPK* gene [25] were obtained from Dr. Gourdon and housed at the CCHMC mouse facility. Genotyping was performed as previously described [22,25] using the following primers: FBF, 5′-TCCTCAGAAGCACTCATCCG-3′; FBWDR, 5′-ACCTCCATCCTTTCAGCACC-3′; and FBFBR, 5′-AACCCTGTATTTGACCCCAG-3′. As controls, WT littermates from the same strain were used. Three-month-old homozygous DMSXL mice of both genders were treated with tideglusib (Sigma) dissolved in labrasol at a dose of 0.1 μg/g of body weight, 2 times a week for 2 weeks. Following drug withdrawal for 11 days, the treatment was continued for an additional 3 weeks in a female group of mice and for 6 weeks in a male group of mice. The grip strength measurements were performed one day before each treatment and the next day after each treatment. CUGBP1-KI mice (CUGBP1 S302A-KI mice) containing CUGBP1 that was unphosphorylatable at Ser333 (human Ser302) by the GSK3β-cyclin D3-CDK4 were described previously [26]. CUGBP1 KI mice were maintained as the heterozygous strain. These mice were genotyped using the following primers: the forward primer, 5′-TTCCTGTTGGCAAGAGAAGGCAAG-3′, and reverse primer, 5′-ATGACAACCAGGGCTTGCCCATTA-3′. 

### 4.2. RNAseq Analysis

Skeletal muscles (gastric) were collected from WT, vehicle and TG-treated *HSA^LR^* mice (0.1 μg/g of body weight for 2 weeks, 2 times a week), as described in [22]. RNA integrity was examined via agarose gel electrophoresis. Extracted RNA was used for RNAseq analysis using OmegaBioservices (Norcross, GA, USA). RNA samples were re-purified using an E.Z.N.A.^R^ total RNA kit (Omega Bio-tek, Norcross, GA) according to the manufacturer’s protocol. The quality of the isolated RNA was verified via calculation of the RNA integrity number using Agilent Technologies. Ribosomal RNAs were depleted from the RNA samples prior to library preparation. Libraries were sequenced with at least 60M reads per sample. Standard bioinformatics differential analysis and pathway analysis were performed using the Rosalind platform https://omegabioservices.rosalind.bio/ (accessed on 20 June 2023).

### 4.3. Western Blot Analysis

Whole-cell proteins were extracted from mouse muscles (gastroc) using RIPA buffer. The quality of the extracted proteins was verified via electrophoretic separation followed by Coomassie staining. Proteins (50 μg) were separated via SDS-polyacrylamide gel electrophoresis, transferred onto a nitrocellulose membrane and analyzed with various antibodies. Antibodies to *Clcn1* (CSBio, Menlo Park, USA); Myf6 (antibodies.com, Cambridge, UK); LAMC2, Sox9, RUNX1 and THBS1 (Thermo Fisher, Cincinnati, OH, USA; TEAD4/TEF3) (Bioss, Woburn, MA, USA); and β-actin (Santa Cruz, Dallas, TX, USA) were used according to the manufacturers’ protocols.

### 4.4. Open Field Test (OFT)

This test was performed using the Open Field Superflex box (Fusion software, v5.3) (Unitech Electronics, Columbus, OH, USA). Care was taken to reduce mouse stress before and during the test. Naïve mice were placed in the box and their movements were analyzed for 5 min to determine various parameters of motor activity and behavior, including stereotypy time, stereotypic activity count, stereotypic episode count, stereotypic episode activity count, vertical movement time, vertical activity count, vertical episode count and left-front time legacy.

### 4.5. Grip Strength Analysis 

The grip strength of the front paws was measured using a grip strength meter (Columbus Instruments, Columbus, OH, USA) as described in [20,21,22]. An average of five measurements were presented.

### 4.6. Magnetic Resonance Imaging and Diffusion Tensor Imaging 

Magnetic resonance imaging and diffusion tensor imaging were performed using a Bruker 7T small animal MRI system and a 38 mm transmit/receive volume coil (Bruker, Billerica, MA, USA). Mice were anesthetized with isoflurane, and then, positioned in the center of the coil. Temperature was maintained at 36–38 °C using a flow of warm air controlled by a Small Animal Instruments monitor (Small Animal Instruments, Inc., Stony Brook, NY, USA). Isoflurane was adjusted to maintain a respiration rate between 40 and 80 breaths per minute. Three-dimensional volumetric data were acquired using a fast spin-echo sequence with a repetition time (TR) of 1800 MS, an echo time (TE) of 10 MS, an echo train length of 20 leading to an effective echo time of 80 MS, a field-of-view (FOV) of 51.2 × 22 × 22 mm^3^, and an acquisition matrix of 256 × 110 × 110 leading to an isotropic 0.2 mm resolution. Diffusion tensor imaging data were then acquired using a spin-echo sequence with an echo planar readout using a TR of 7500 MS, TE of 24 MS, FOV of 25.6 × 25.6 mm^2^, acquisition matrix of 114 × 114, in-plane resolution of 0.225 mm × 0.225 mm, 0.5 mm slice thickness, 5 slices and 6 diffusion directions with a b-value of 850.

Volumetric data were imported into ImageJ, cropped to contain mainly brain, and rescaled by a factor of 10. The cropped and scaled images were brain-extracted and segmented into tissue and CSF using FSL’s BET algorithm [46]. Tissue volume and CSF volume were calculated from the segmented images using *fslstats*, a tool within FSL. DTI data were imported into DTI studio [47], which was used to generate parametric maps (fractional anisotropy, FA; radial diffusivity, RD; axial diffusivity, AD; and mean diffusivity, MD). The maps were saved and imported into ImageJ to draw regions of interest in the corpus callosum, left and right external capsule, left and right fimbria and left and right internal capsule.

### 4.7. Statistical Analysis

The values were analyzed via the Student’s *t*-test using SPSS statistics. The results were considered significant if the *p* value was <0.05.

## Figures and Tables

**Table 1:** The total number of genes (up- and down-regulated) altered in skeletal muscle (gastroc) in the vehicle-treated *HSA^LR^* mice vs. WT mice and in the TG-treated *HSA^LR^* mice vs. the vehicle-treated *HSA^LR^* mice is shown.

	Number of Altered Genes	Number of Altered Genes, Improved by 2-Week Treatment by TG 115	Percent of Genes, Normalized by TG
Total altered genes (>1.5 fold) in *HSA*^LR^ muscle (gastroc) vs WT	665	117	~17%
Total altered genes (>1.5 fold) in TG-treated *HSA*^LR^ muscle (gastroc) vs vehicle-treated *HSA*^LR^ muscle	675		

**Table 2:** The most significant enriched down-regulated processes in the skeletal muscle (gastroc) of the vehicle-treated *HSA^LR^* mice vs. WT mice are shown. The number of genes improved by the TG treatment is also shown. ^a^ Genes selected for confirmation via Western blot analysis. ^b^ Genes altered in *HSA^LR^* mice were examined in skeletal muscle (gastroc) of matched homozygous CUGBP1-KI mice in which CUGBP1 was un-phosphorylatable at S302 [26]. The full names of the shown genes are as follows: Acvr2b (**activin A type IIB receptor**), Akap5 (A-Kinase Anchoring Protein 5), Aldoc (Aldolase, Fructose-Bisphosphate C), Angptl4 (Angiopoietin-Like 4), Atf3 (Activating Transcription Factor 3), Avil (Advillin)**,** Baiap2 (Brain-Specific Angiogenesis Inhibitor I-Associated Protein 2), Cdnf (Cerebral Dopamine Neurotrophic Factor), Cd27 (CD27 Antigen or Tumor Necrosis Factor Receptor Superfamily, Member 7), Chodl (Chondrolectin), *Clcn1* (chloride channel 1), **Crhr2** (**corticotropin-releasing hormone receptor 2**), Dlg5 (Discs Large MAGUK Scaffold Protein 5), Dpp6 (Dipeptidyl aminopeptidase-like protein 6), Esr2 (Estrogen Receptor 2), Fgb (fibrinogen B), Fscn2 (Fascin Actin-Bundling Protein 2), Fzd7 (Frizzled Class Receptor 7), Hbegf (Heparin-Binding EGF-Like Growth Factor), Hspb1 (Heat Shock Protein Family B (Small) Member 1), Kdr (Kinase Insert Domain Receptor); Klhl40 (Kelch-Like Family Member 40), Lamc2 (Laminin Subunit Gamma 2), Lrrc 38 (Leucine-Rich Repeat-Containing 38 or BK Channel Auxilliary Gamma Subunit LRRC38), Mug1 (Murinoglobulin 1), Myc (MYC Proto-Oncogene), Myf6 (Myogenic factor 6), Nes (Nestin), Nrxn1 (Neurexin-1), Pak1 (P21 (RAC1) Activated Kinase 1), Park7 (Parkinsonism-Associated Deglycase), Pcp4 (Purkinje Cell Protein 4), Peg10 (Paternally Expressed 10), Pla2g12a (Phospholipase A2 Group XIIA), Plk2 (Polo-Like Kinase 2), Rab3c (RAB3C, Member of RAS Oncogene Family), Rgcc (Regulator of Cell Cycle), Rrad (Ras-Related Glycolysis Inhibitor And Calcium Channel Regulator), Runx1 (Runt-Related Transcription Factor 1), Sh3pxd2b (SH3 and PX Domains 2B), Snap25 (Synaptosome-Associated Protein 25), Slc1a2 (Solute Carrier Family 1 Member 2), Sox9 (SRY-Box Transcription Factor 9), Sptbn2 (Beta-III Spectrin), Stxbp4 (Syntaxin-Binding Protein 4), Syt9, 12 and 14 (Synaptotagmins 9, 12 and 14), Tead4 (TEA Domain Transcription Factor 4), Thbs1 (Thrombospondin 1), Tmem100 (Transmembrane Protein 100), Tmem120b (Transmembrane Protein 120B), Tnfrsf12 (Tumor Necrosis Factor Ligand Superfamily, Member 12), Trp73 (Tumor Protein P73), Vmp1 (Vacuole Membrane Protein 1), Xbp1 (X-Box Binding Protein 1), Xirp1 (Xin Actin Binding Repeat-Containing 1).

Pathway	Altered Genes (up and down) in *HSA*^LR^ vs WT Muscle (n)	*p* Value	Genes, Improved by TG in *HSA*^LR^ Muscle (n)	Genes Improved in TG-Treated *HSA*^LR^ Muscle
Regulation of hormone levels	35	1.4 × 10^−7^ (down); 0.1365 (up)	7	Snap25, Fgb, Acvr2b, Crhr2, Nrxn1,Stxbp4, Runx1 ^a,b^
Regulation of transport	93	1 × 10^−6^ (down); 0.001689 (up)	10	Slc1a2, Stxbp4, Pop4, *Clcn1* ^a^, Snap25, Dpp6, Fgb, Rgcc, Runx1 ^a,b^, Rrad
Secretion	30	1.26 × 10^−6^ (down); 0.368332 (up)	14	Snap25, Acvr2b, Sptbn2, Pla2g12a, Myc, Pak1, Vmp1, Syt14, Syt9, Syt12, Park7, Xbp1, Cd27, Trp73
Synaptic vesicle localization	12	1.5 × 10^−6^ (down); 0.328787 (up)	2	Snap25, Sptbn2
Synaptic vesicle cycle	14	1.75 × 10^−6^ (down); 0.0558 (up)	3	Sptbn2, Snap25, Nrxn1
System development	185	1.8 × 10^−6^ (down); 6.74 × 10^−6^ (up)	34	Snap25, Avil, Nrxn1, Pcp4, Crhr2, Acvr2b, Kdr; Fzd7, Cdnf, Sptbn2, Akap5, Slcla2, Angptl4, Sh3pxd2b, Tnfrsf12, Sox9 ^a,b^, Atf3, Chodl, Hbegf, Tmem100, Esr2, Klhl40, Fscn2, Xirp1, Plk2, Baiap2, Thbs1 ^a,b^, Hspb1, Dlg5, Tead4 ^a^, Lamc2 ^a,b^, Peg10, Runx1, Myf6 ^a,b^
Secretion by cell	24	3.88 × 10^−6^ (down); 0.4887 (up)	6	Akap5, Nrxn1, Acvr2b, Sptbn2, Snap25, Rab3c
Developmental Process	223	6.39 × 10^−6^ (down); 1.35 × 10^−5^ (up)	32	Aldoc, Sptbn2, Mug1, Slc1a2, 20Akap5, Avil, Snap25, Pcp4, Nrxn1, Acvr2b, Crhr2, Lrrc38, Fzd7, Kdr, Hspb1, Baiap2, Runx1 ^a,b^, Peg10, Tead4, Dlg5, Myf6 ^a,b^,Tnfrsf12, Sh3pxd2 ^b^, Chodl, Atf3, Sox9 ^a,b^, Nes, Xirp1, Klhl40, Fscn2, Esr2, Tmem100
Cell differentiation	164	3.89 × 10^−6^ (down); 2.36 × 10^−6^ (up)	20	Pcp4, Cdnf, Avil, Snap25, Fzd7, Kdr, Crhr2, Acvr2b; Myf6 ^a,b^, Peg10, Runx1 ^a,b^, Dlg5, Tead4 ^a^, Baiap2, Tmem120b, Klh140, Tmem100, Esr2, Atf3, Sox9 ^a,b^

**Table 3:** The percentage and survival rate of hetCUGBP1-KI-hetDMSXL, WT-CUGBP1-KI-homDMSXL and homCUGBP1-KI-homDMSXL mice in the double-mutant CUGBP1-KI-DMSXL strain is shown. The analysis is based on 23 crosses of heterozygous CUGBP1-KI mice with heterozygous DMSXL mice.

Genotype	Born Mice Number (23 crosses, 232 pups)	%	Survival Rate, %
hetKI-hetDMSXL	56	24.1	94.6
WTKI-homDMSXL	28	12.1	71.4
homKI-homDMSXL	12	5.15	41.7

## Data Availability

The data are contained within this article. The GEO submission number for the RNAseq data is GSE230821.
